# Short-term immunogenicity of standard and accelerated hepatitis B virus vaccination schedules in healthy adults: a comparative field study in China

**DOI:** 10.1042/BSR20180846

**Published:** 2018-10-15

**Authors:** Xuan Zhang, Juan Wang, Xi Chen, Menglu Yu, Shuangbin Yu, Yuanyuan Sun, Jinchao Duan, Hongying Sun, Ping Yuan

**Affiliations:** 1 Department of Epidemiology and Statistics, West China School of Public Health, Sichuan University, Chengdu 610041, Sichuan Province, China; 2 Center of Infectious Disease, West China Hospital, Sichuan University, Chengdu 610041, Sichuan Province, China; 3 Center for Disease Control and Prevention of Mianyang, Mianyang 621000, Sichuan Province, China

**Keywords:** accelerated schedule, Hepatitis B virus, hepatitis B vaccine, immunization program, standard schedule

## Abstract

World Health Organization recommends hepatitis B virus (HBV) immunization at 0, 1, and 6 months. However, studies have suggested that shortening the interval between the first and last HBV immunization can improve completion rates. Less clear is whether accelerated immunization is as immunogenic as standard immunization. Thus, the present study aimed to compare the short-term immunogenicity of yeast-derived hepatitis B vaccine in healthy adults immunized on an accelerated or standard schedule. Between June 2013 and March 2014, individuals from Jinfeng and Longmen, China were randomly assigned to receive the vaccine on an accelerated schedule (at 0, 1, and 2 months; *n*=201) or a standard schedule (at 0, 1, and 6 months; *n*=206). Subjects filled out a questionnaire asking about demographic and other health data, and they underwent physical examination. Blood was assayed for HBV surface antigen and HBV surface antibody (HBsAb) at 1–2 months after the three-dose schedule. Multivariate binary logistic regression was used to determine whether the rate of anti-HBs seroconversion differed with immunization schedule. Covariance analysis was used to compare geometric mean HBsAb concentration between the two schedules. The anti-HBs seroconversion rate was 84.6% in the accelerated group and 90.3% in the standard group. After controlling for several potential confounders, the accelerated schedule was associated with significantly lower anti-HBs seroconversion rate (OR: 0.560, 95% CI: 0.318–0.988). Similarly, the accelerated schedule was associated with significantly lower geometric mean HBsAb concentration. These results suggest that the standard schedule is more likely to lead to anti-HBs seroconversion and higher HBsAb levels in adults.

## Introduction

Incidence of hepatitis B virus (HBV) in China among children younger than 15 years fell significantly after 1992, when the Ministry of Health included HBV vaccination in the national immunization program [1]. However, HBV incidence among adults remains substantial. A national survey in China showed that 8.57% of adults aged 15–59 years are positive for HBV surface antigen (HBsAg), and 47.38% of adults are positive for anti-HBV surface antibodies (HBsAb) [2].

HBV vaccination is considered the safest, most effective way to prevent HBV infection [3–5]. The World Health Organization and US Centers for Disease Control and Prevention, as well as Chinese National Guidelines on chronic hepatitis B prevention and treatment (2015) recommend HBV immunization at 0, 1, and 6 months. This long duration translates to low completion rates [6] and has contributed to the fact that in the US only 24.5% of adults aged 19 and older were vaccinated against HBV in 2014 [7]; in China, the adult vaccination rate is below 10%, according to a 2006 national HBV seroprevalence survey [8].

Studies have suggested that shortening the interval between the first and last HBV immunization can improve completion rates and even stimulate earlier and faster HBsAb production [9,10]. For example, an accelerated immunization schedule can encourage injected drug users to complete HBV vaccination [11], and it can increase the acceptability of vaccination among the general population [12].

Less clear, however, is whether accelerated immunization is as immunogenic as standard immunization. One study, for example, showed that although 93% of individuals vaccinated on an accelerated schedule achieved the desired HBsAb titer within 1 month after the third injection, the rate of individual’s positive for such antibodies as well as the titer of such antibodies tended to decrease after 12 months [13]. Given the attractiveness of accelerated HBV immunization, whether it is as effective at eliciting antibody production as the standard immunization needs to be further studied.

Therefore, the present epidemiological field study was undertaken to compare the immunogenicity of an accelerated HBV vaccination schedule (at 0, 1, and 2 months) with the standard schedule (at 0, 1, and 6 months).

## Methods

### Setting

The towns of Jinfeng and Longmen were randomly selected from among the 21 towns and subdistricts in Mianyang, China covered by the National Science & Technology Pillar Program of the 12th Five-Year Plan. Eligible subjects in the towns were enrolled and randomly assigned to receive accelerated HBV vaccination (at 0, 1, and 2 months) or standard vaccination (at 0, 1, and 6 months). In the end, subjects in Jinfeng received the accelerated schedule, while subjects in Longmen received the standard schedule. The same vaccine and dose were used in all cases.

### Eligibility criteria

Study subjects were recruited from the cohort of individuals enrolled in the National Science & Technology Pillar Program during the 12th Five-Year Plan. This cohort contained individuals from Mianyang City aged 15–59 years who were negative for HBsAg and HBsAb at the time of enrollment.

The present study was carried out from 1 June 2013 to 1 March 2014.

Individuals were eligible to participate in the study if they (1) were negative for HBsAg and HBsAb, (2) were 15–59 years old, (3) had lived in Mianyang City longer than 6 months by the time of enrollment, and (4) voluntarily consented to participate in the study. The study was approved by the Ethics Committee of West China Hospital, Sichuan University. All subjects gave written informed consent to receive HBV vaccination and to participate in follow-up.

Subjects were excluded from the study if they (1) were positive for HBsAg or HBsAb, (2) had a history of serious vaccine reaction, (3) were known to have immune dysfunction or were considered susceptible to it, (4) were currently on immunosuppressive therapy, (5) had experienced fever (>38°C) during 3 days prior to enrollment, or (6) had been vaccinated with live attenuated virus during the preceding month, such as measles (including MMR, and leprosy), Japanese encephalitis virus, hepatitis A virus, or varicella zoster virus.

### Vaccination and data collection

Research staff received appropriate training from the lead project investigators, and then collected data at examination centers in local health stations and community clinics in Jinfeng and Longmen. Staff conducted standardized, questionnaire-based face-to-face interviews after obtaining written consent from participants. The questionnaire collected information about demographics (including gender, age, height, and weight), health-related behaviors (smoking and drinking), family history of hepatitis B (parents infected by HBV or not), and other data. Each questionnaire was assigned a unique identification number.

Subjects who, upon screening, showed no serological evidence of past HBV infection or immunization were offered vaccination through the regular service of township hospitals, where trained medical stuff performed vaccinations by intramuscular injection. The vaccine (Hualan Biological Vaccine Company, Chengdu, China) contained 10 μg recombinant HBsAg per dose.

All subjects were tested before and after vaccination for the presence of HBsAg, anti-HBs, and anti-HBc. Testing was performed at an external laboratory using commercially available kits (Sichuan Kingmed Center for Clinical Laboratory, Chengdu, China). At 1–2 months after the third vaccine injection, subjects’ blood was sampled (5 ml) and assayed for anti-HBs seroconversion.

### Blood sample tests

The chemiluminescence microparticle immunoassay was used to detect HBV serum markers (ARCHITET i2000, Abbott, U.S.A.). Seroprotection was defined as HBsAg ≥ 0.05 IU/ml, an anti-HBs titer ≥10 IU/l, and anti-HBc (s/co) ≥1 at 1–2 months after the third vaccination. The immune response of Hep B was classified based on anti-HBs concentration as none (<10 IU/l) [13], low (10–100 IU/l) [14], normal (100–1000 IU/l), or high (≥1000 IU/l).

### Statistical analysis

SPSS 22.0 (IBM, Chicago, IL, U.S.A.) was used for all statistical analyses, with significance defined as *P*<0.05. Subjects on accelerated or standard vaccination schedules were compared in terms of demographic characteristics, health-related behaviors, family history of hepatitis B, and anti-HBs seroconversion rate. Differences were assessed for significance using the Pearson χ^2^ and Fisher’s exact tests. Multivariate binary logistic regression was used to compare anti-HBs seroconversion rates between the two vaccination schedules after controlling for age, gender, body mass index (BMI), smoking, drinking, anti-HBc, and family history of hepatitis B.

Anti-HBs concentrations were log-transformed to give them a normal distribution, and then used to calculate anti-HBs geometric mean concentration (GMC), which was compared between subjects on accelerated and standard vaccination schedules using the *t* test. GMC was also compared between subgroups of subjects stratified by different characteristics, and differences were assessed for significance using the *t* test, ANOVA, and Scheffe test. Finally, covariance analysis was used to compare anti-HBs GMC between the accelerated and standard schedules after controlling for various potential confounders.

### Ethical approval

The present study was approved by the Ethics Committee of West China Hospital, Sichuan University, and it conformed to the provisions of the Declaration of Helsinki. Each participant signed an informed consent form before enrollment.

## Results

### Study population

Between 1 June 2013 and 1 March 2014, 407 individuals underwent blood testing and were allocated to undergo vaccination on the accelerated schedule (201, 49.39%) or standard schedule (206, 50.61%). The remaining eligible individuals did not consent to participate in the study.

Subjects on the accelerated schedule (36.3% men) had an average age of 38.1 ± 12.8 years and average BMI of 23.0 ± 3.6 kg/m^2^. Subjects on the standard schedule (38.8% men) had an average age of 39.7 ± 11.8 years and average BMI of 23.0 ± 3.5 kg/m^2^. The two groups were similar in age, gender, BMI, smoking, drinking, anti-HBc, and family history of hepatitis B (*P*>0.05, Table 1).

**Table 1:** Characteristics of the study population

Variable		Accelerated schedule (*n*=201)	Standard schedule (*n*=206)	χ^2^/*u*	*P*
Age, year	15–29	60 (29.9)	52 (25.3)	2.891	0.236
	30–49	89 (44.3)	107 (51.9)		
	50–59	52 (25.9)	47 (22.8)		
Sex	Male	73 (36.3)	80 (38.8)	0.327	0.568
	Female	128 (63.7)	126 (61.2)		
BMI, kg/m^2^		23.0 ± 3.6	23.0 ± 3.5	0.022	0.983
Smoking	Yes	33 (16.4)	44 (21.4)	1.819	0.177
	No	168 (83.6)	162 (78.6)		
Drinking, g/day	No	149 (74.2)	149 (72.3)	1.588	0.452
	<20	25 (12.4)	33 (16.0)		
	≥20	27 (13.4)	24 (11.7)		
Family history of hepatitis B	Yes	3 (1.5)	2 (1.0)	0.230	0.631
	No	198 (98.5)	204 (99.0)		
Anti-HBc	Negative	110 (54.7)	112 (54.4)	0.005	0.941
	Positive	91 (45.3)	94 (45.6)		

Values are *n* ± S.D. or *n* (%), unless otherwise noted.

### Rate of anti-HBs seroconversion

**Figure 1:**
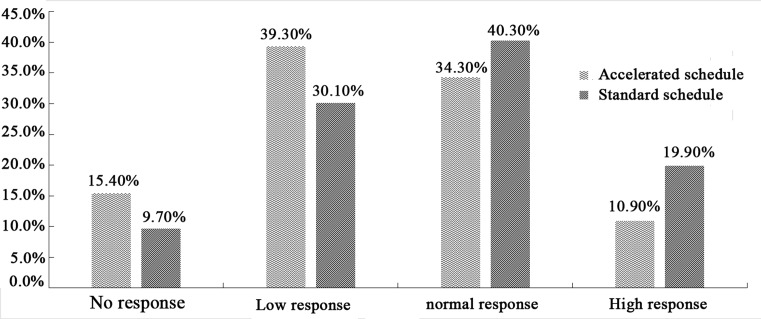
Anti-HBs seroconversion rates on the two vaccination schedules The left vertical axis represents the rate of anti-HBs seroconversion. Wave represents the anti-HBs seroconversion rates on accelerated schedule in each group, and slash represents the anti-HBs seroconversion rates on standard schedule in each group. The horizontal axis represents the reaction type.

**Figure 2:**
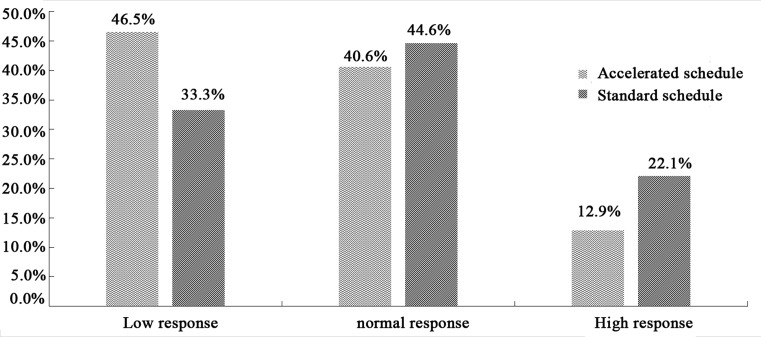
Anti-HBs seroconversion rates on the two vaccination schedules among subjects who produced anti-HBs The left vertical axis represents the constituent ratio. Wave represents the constituent ratio on accelerated schedule in each group, and slash represents the constituent ratio on standard schedule in each group. The horizontal axis represents the reaction type among subjects who produced anti-HBs.

The rate of anti-HBs seroconversion was 84.6% in the accelerated group and 90.3% in the standard group. The proportion of subjects showing low response to HBV vaccination was higher in the accelerated group (39.3%) than in the standard group (30.1%). Conversely, lower proportions of subjects in the accelerated group showed normal response (34.3% vs 40.3%) or high response (10.9% vs 19.9%; Figure 1). Among subjects on the accelerated schedule who produced anti-HBs, 46.5% showed low response, 40.6% normal response, and 12.9% high response. The corresponding proportions among subjects on the standard schedule who produced anti-HBs were 33.3%, 44.6%, and 22.1% (Figure 2).

**Table 2:** Univariate analysis to identify variables associated with anti-HBs seroconversion rate on the two vaccination schedules

Variable		Accelerated schedule	Standard schedule	χ^2^	*P*
		Seroconversion % (*n/N*)	Seroconversion % (*n/N*)		
Age, year	15–29	93.3 (56/60)	94.2 (49/52)		1.000^†^
	30–49	85.4 (76/89)	90.7 (97/107)	1.737	0.187
	50–59	73.1 (38/52)	85.1 (40/47)	2.617	0.106
	χ^2^	8.620	3.747		
	*P*	0.003*	0.053*		
Sex	Male	79.5 (58/73)	90.0 (72/80)	4.520	0.034
	Female	87.5 (112/128)	90.5 (114/126)	0.384	0.759
	χ^2^	2.308	0.002		
	*P*	0.129	0.960		
BMI	<18.5	100 (20/20)	95.0 (19/20)		1.000^†^
	18.5–23.9	89.2 (91/102)	92.8 (103/111)	1.203	0.273
	24–27.9	75.0 (45/60)	89.8 (53/59)	5.289	0.021
	≥28	73.7 (14/19)	68.8 (11/16)	0.043	0.836
	χ^2^	10.206	9.866		
	*P*	0.001*	0.002*		
Smoking	Yes	78.8 (26/33)	86.4 (38/44)	3.833	0.052
	No	85.7 (144/168)	92.1 (116/126)	0.748	0.387
	χ^2^	1.014	2.177		
	*P*	0.314	0.140		
Drinking					
(g/day)	No	86.6 (129/149)	91.3 (136/149)	2.354	0.125
	<20	80.0 (20/25)	87.9 (29/33)		0.494^†^
	≥20	77.8 (21/27)	87.5 (21/24)		0.315^†^
	*P*	0.335^†^	0.551^†^		
Anti-HBc	Negative	90.0 (99/110)	93.8 (105/112)	1.312	0.252
	Positive	78.0 (71/91)	86.2 (81/94)	2.904	0.088
	χ^2^	5.478	4.349		
	*P*	0.019	0.037		
Family history of hepatitis B	Yes	100 (3/3)	100 (2/2)		
	No	84.3 (167/198)	90.2 (184/204)	4.069	0.044
	*P*	1.000^†^	1.000		

* Cochran–Armitage trend test.

† Fisher’s exact test.

Univariate analyses revealed a significantly lower rate of anti-HBs seroconversion among men on the accelerated schedule than among men on the standard schedule (χ^2^ = 4.520, *P*<0.05; Table 2). Subjects with BMI between 24 and 28 showed a significantly lower anti-HBs seroconversion rate on the accelerated schedule than on the standard one (χ^2^ = 5.289, *P*<0.05). Similarly, the accelerated schedule was associated with significantly lower anti-HBs seroconversion rate among subjects without a family history of hepatitis B (χ^2^ = 4.069, *P*<0.05). The following factors were negatively associated with anti-HBs seroconversion rate: BMI below 18.5, between 18.5 and 24, or above 28; age; female gender; smoking; drinking; anti-HBc; and family history of hepatitis B (Table 2).

**Table 3:** Univariate analysis to identify variables associated with anti-HBs GMC on the two vaccination schedules

Variable		Anti-HBs GMC (mIU/ml)		*t*	*P*
		Accelerated schedule	Standard schedule		
Age, year	15–29	96.107	287.268	4.101	<0.001
	30–49	78.686	137.155	2.401	0.017
	50–59	47.023	65.406	1.037	0.302
	*F*	2.483	14.411		
	*P*	0.086	<0.001		
Sex	Male	70.996	129.432	2.243	0.026
	Female	74.472	147.383	3.489	0.001
	*t*	0.185	0.636		
	*P*	0.853	0.525		
BMI	<18.5	160.405	288.402	1.266	0.212
	18.5–23.9	76.325	165.687	3.828	<0.001
	24–27.9	48.420	114.950	2.969	0.004
	≥28	92.842	39.210	1.275	0.209
	*F*	2.663	7.308		
	*P*	0.049	<0.001		
Smoking	Yes	52.288	93.874	1.565	0.121
	No	77.799	156.088	4.003	<0.001
	*t*	1.343	2.097		
	*P*	0.181	0.037		
Drinking					
(g/day)	No	78.910	147.727	3.463	0.001
	<20	60.734	147.383	2.023	0.047
	≥20	57.306	96.494	1.022	0.311
	*F*	1.012	0.539		
	*P*	0.365	0.584		
Anti-HBc	Negative	86.044	220.485	4.740	<0.001
	Positive	60.130	81.311	1.249	0.213
	*t*	1.448	5.203		
	*P*	0.149	<0.001		
Family history of hepatitis B	Yes	246.776	345.776	0.475	0.660
	No	71.841	138.864	4.133	<0.001
	*t*	1.228	0.923		
	*P*	0.221	0.357		

Multivariate analyses showed that, after controlling for age, gender, BMI, smoking, drinking, anti-HBc, and family history of hepatitis B, the anti-HBs seroconversion rate was significantly lower for the accelerated schedule than for the standard one (odds ratio [OR]: 0.560, 95% confidence interval [CI]: 0.318–0.988).

### Anti-HBs GMC

Anti-HBs GMC was 73.197 mIU/ml (95% CI: 57.320–95.302) in the accelerated group and 140.134 mIU/ml (95% CI: 115.193–170.425) in the standard group.

Univariate analysis indicated that for subjects aged 15–29 or 30–49 years, the accelerated schedule was associated with significantly lower anti-HBs GMC than the standard schedule (Table 3). The accelerated schedule was also associated with significantly lower anti-HBs GMC than the standard schedule for the following subgroups: men, women, non-smokers, subjects with BMI between 18.5 and 24, or between 24 and 28, subjects who drink <20 g/day, subjects positive for anti-HBc, and subjects without a family history of hepatitis B.

**Table 4:** Covariance analysis to compare anti-HBs GMC (mIU/ml) on the two vaccination schedules

Schedule	Raw GMC	Calibrated GMC	Calibrated 95% CI	*F*	*P*
Accelerated (*n*=201)	73.197	72.473	57.429–91.325	19.287	<0.001
Standard (*n*=296)	140.134	141.219	116.850–170.187		

Covariance analysis showed that, after controlling for age, gender, BMI, smoking, drinking, anti-HBc, and family history of hepatitis B, the calibrated anti-HBs GMC was 72.473 mIU/ml (95% CI: 57.429–91.325) in the accelerated group and 141.219 mIU/ml (95% CI: 116.850–170.187) in the standard group; the calibrated value was significantly lower in the accelerated group (*F* = 19.287, *P*<0.001; Table 4).

## Discussion

The present study shows that, after controlling for age, sex, BMI, smoking, drinking, anti-HBc, and family history of hepatitis B, an accelerated HBV immunization schedule was associated with significantly lower anti-HBs seroconversion rate than the standard immunization schedule (OR: 0.560, 95% CI: 0.318–0.988). These results are consistent with previous work in a different region of China [15]. Similarly, we found by covariance analysis that the accelerated schedule was associated with significantly lower anti-HBs GMC (*F* = 19.287, *P*<0.001). Our results are consistent with several studies reporting lower immunogenicity of accelerated vaccination schedules [16–21]. This may be explained by the shorter interval between the second and third dose in the accelerated schedule [21].

The higher frequency of low vaccination response in our accelerated group than in the standard group suggests that the standard schedule stimulates an immune response more easily [22]. Studies suggest that individuals usually possess long-term immunity to HBV if anti-HBs concentration is at least 100 mIU/ml. Our results suggest that the standard schedule is better at ensuring minimum immune response levels for sustained protective effects.

Consistent with this, we found that only 84.6% of subjects on the accelerated schedule developed anti-HBs titer ≥10 mIU/ml, which is considered the minimum needed to withstand HBV infection [23]. In contrast, 90.3% of subjects on the standard schedule achieved this minimum. A higher percentage of subjects on an accelerated schedule (93.6%) achieved this minimum in a study in South Korea [24], which may reflect the fact that the vaccine in that study was delivered subcutaneously and contained 0.15 ml recombinant HBsAg per dose. Administering a higher dose subcutaneously may enhance immune responses to HBV [25,26].

Despite the apparent superiority of the standard schedule, both the standard and accelerated schedules achieved anti-HBs seroconversion rates above 80%, which compares well with the rates reported for healthy adults on a standard schedule [27,28]. Our results add to the evidence base showing that HBV vaccination can elicit protective immune responses in healthy adults.

One potential benefit of an accelerated vaccination schedule is earlier protection even before the series of injections is complete [29–31]. In fact, one study examined anti-HBV immune responses at 2 years after vaccination on an accelerated or standard schedule and found lower HBV infection prevalence among those immunized on an accelerated schedule [32]. One possible explanation is that subjects on the standard schedule are at greater risk of suffering infection before they complete the vaccination course. This implies that individuals who urgently require protection from HBV may benefit from the accelerated schedule. It may also benefit individuals who, for various reasons, are at higher risk of failing to complete the lengthy standard schedule [33].

The results of the present study should be interpreted with caution in light of several limitations. First, we convenience-sampled residents of only two towns in one region of China, which increases the risk of bias in our study. We collected data only on anti-HBs response at 1–2 months after the third injection, preventing us from comparing the long-term immune effects of the two vaccination schedules.

## Conclusion

More than 80% of subjects vaccinated on a standard or accelerated schedule presented anti-HBs concentrations above 10 mIU/ml at 1–2 months after the last vaccination. This highlights the usefulness of vaccinating adults against HBV. The accelerated schedule was associated with lower anti-HBs seroconversion rate and lower anti-HBs GMC than the standard schedule. Therefore, the standard schedule may be more effective for eliciting anti-HBV immune protection.

## Abbreviations

BMI: body mass index
CI: confidence interval
GMC: geometric mean concentration
HBsAb: HBV surface antibody
HBsAg: HBV surface antigen
HBV: hepatitis B virus
OR: odds ratio
